# Fetal Lung-Derived Exosomes in Term Labor Amniotic Fluid Induce Amniotic Membrane Senescence

**DOI:** 10.3389/fcell.2022.889861

**Published:** 2022-07-04

**Authors:** Shuting Wan, Pengzheng Chen, Mengqi Gu, Jing Liu, Qian Zhou, Fengyuan Zhang, Yuan Lu, Lei Li, Xietong Wang

**Affiliations:** ^1^ Department of Obstetrics and Gynaecology, Shandong Provincial Hospital Affiliated with Shandong University, Jinan, China; ^2^ Department of Obstetrics and Gynaecology, Shandong Provincial Hospital Affiliated with Shandong First Medical University, Jinan, China; ^3^ The Laboratory of Medical Science and Technology Innovation Center (Institute of Translational Medicine), Shandong First Medical University (Shandong Academy of Medical Sciences) of China, Jinan, China; ^4^ Department of Obstetrics and Gynaecology, Maternal and Child Health Care of Shandong Province, Jinan, China; ^5^ The Laboratory of Placenta-Related Diseases, Key Laboratory of Birth Regulation and Control Technology of the National Health and Family Planning Commission of China, Jinan, China

**Keywords:** parturition, exosome, inflammation, senescence, fetal lung

## Abstract

The mechanism of parturition is still unclear. Evidence has shown that delivery is associated with cellular senescence of the amniotic membrane. We isolated fetal lung-associated exosomes from the amniotic fluid from term labor (TL-exos) and verified that the exosomes can cause primary human amniotic epithelial cell (hAEC) senescence and apoptosis and can release higher levels of senescence-associated secretory phenotype (SASP)-related molecules and proinflammatory damage-associated molecular patterns (DAMPs) than exosomes isolated from the amniotic fluid from term not in labor (TNIL-exos). The human lung carcinoma cell lines (A549) can be used as an alternative to alveolar type 2 epithelial cells producing pulmonary surfactant. Therefore, we isolated A549 cell-derived exosomes (A549-exos) and found that they can trigger hAEC to undergo the same aging process. Finally, the animal experiments suggested that A549-exos induced vaginal bleeding and preterm labor in pregnant mice. Therefore, we conclude that exosomes derived from fetal lungs in term labor amniotic fluid induce amniotic membrane senescence, which may provide new insight into the mechanism of delivery.

## Introduction

Under normal circumstances, the fetus matures after 37 weeks, the amniotic membrane ages, and labor starts. However, the mechanisms promoting enhanced uterine contraction and the initiation of parturition are still unclear. Multiple researches show that inflammatory oxidative reactions being a result of multifactorial regulation are associated with labor. By inflating the intrauterine balloon, labor was induced in non-human primates, leading to increased levels of IL-6, IL-8, and CCL- (Adams Waldorf et al., 2015) in the myometrium. Thus, enhanced inflammation resulting from mechanical stress induce the onset of term labor. In early pregnancy, progesterone/progesterone receptor (P4/PR) inhibits the pro-inflammatory transcription factors nuclear factor kB (NF-kB) and activating protein 1 (AP-1) transcriptional activity to maintains myometrial quiescence (Peng et al., 2018). However, P4 declines and estrogen (E) becomes dominant near term, and contraction-associated proteins (CAPs) become expressed, including receptors for oxytocin and prostaglandins (PGs) (Ilicic et al., 2020). An ehhanced estrogen receptor alpha (ERa) activity in the myometrium precedes the increase in uterine contractility. As well as causing migration of immune cells to the uterus, estrogens antagonize the inflammatory effects of P4 and PR (Hadley et al., 2018a; Amini et al., 2019). Another mechanism of parturition is associated with exosome. Exosomes from primary human epithial cell can cause increased level of interleukin-6 (IL-6), interleukin-8 (IL-8), and PGE2 and activation of NF-kB of myometrial and decidual cells. Therefore the exsome plays an important role in inflammatory response of maternal uterine cells, which induce labor-promoting changes (Robertson et al., 2018).

The unique process of pregnancy is associated immunity, and normal pregnancy rely on a series of inflammatory reactions. A pro-inflammatory state is significant to enhances implantation and initiate labor in the first and third trimesters (Robertson et al., 2018), respectively and an anti-inflammatory state facilitates fetal growth in the second trimester (Mor et al., 2011). During the implantation (inflammation) period, 50–70% of decidua lymphocytes are NK cells, which are also called decidual natural killer (dNK) cells. It can release cytokines and chemokines, including IL-15, IL-6, IL-8, CXCL10, and CXCL11 (Zhang and Wei, 2021). And a large number of decidual immune cells are activated and recruited to the endometrium by those pro-inflammatory factors at the window of implantation. All events are helpful to trophoblast invasion, vascular remodeling and embryonic implantation (Burton and Jauniaux, 2015). Following successful implantation, the second cruial stage of gestation is initiated: the placenta and fetus growth rapidly. And anti-inflammatory factors dominate the second trimester. The maternal–fetal interface connecting mother and fetal is composed of placenta (Ferreira et al., 2017), and dNK cells at this interface play an important role in maintaining pregnancy. Human leukocyte antigen (HLA) ligands (e.g., HLA-G, HLA-C and HLA-E) expressed on extravillous trophoblast (EVT) can interact with dNK cells to inhibit the cytotoxicity of dNK cells to support gestation (Godin-Ethier et al., 2011). Another mechanism related to immune tolerance is that Indoleamine 2,3-dioxygenase (IDO), a cruial metabolic enzyme, is used to degrade tryptophan (Menon et al., 2020). Evidence show that the fetal–maternal interface contain a high level of IDO, which may contribute to decrease dNK-cell cytotoxicity and have a vital influence on maintaining normal pregnancy (Burton and Jauniaux, 2015)^.^ During late pregnancy, delivery occurs because tolerance of the maternal–fetal interface is disrupted, and proinflammatory factors dominate (Cha and Aronoff, 2017; Robertson et al., 2018). Uterine tissue undergoes a phasic transformation at the end of gestation from relative quiescence to maintain pregnancy to a uterine active phase, which prepares for labor and produces stimulatory molecules causing labor onset. In the third trimester of pregnancy, the growth of fetus and the increase of amniotic fluid lead to the passive mechanical stretching of uterine tissue, which may be one of the reasons to stimulate the occurrence of labor. In comparison to singleton pregnancies, twin and multiple pregnancies are associated with a higher rate of preterm birth, which may be caused by uterine overdistension. Compared with women who were not in labor, monocyte chemoattractant protein-1/C-C motif ligand 2 (MCP-1/CCL2), a β-chemokine that attracts and activates macrophages, was found to be increased in the term pregnant myometrium of women in labor (Boros-Rausch et al., 2021).

During late pregnancy, delivery occurs because tolerance of the maternal–fetal interface is disrupted, and proinflammatory factors dominate (Hadley et al., 2018a; Menon et al., 2020). Uterine tissue undergoes a phasic transformation at the end of gestation from relative quiescence to maintain pregnancy to a uterine active phase, which prepares for labor and produces stimulatory molecules causing labor onset. Before term delivery, increased oxidative stress causes inflammation, which induces foetal membrane senescence during this process (Polettini et al., 2015; Cha and Aronoff, 2017; Lavu et al., 2020). The aging cells can release higher level of particular inflammatory markers of SASP, including IL-6, IL-8, tumour necrosis factor-alpha (TNF-α), metalloproteinases (MMPs), and granulocyte-macrophage colony-stimulating factor (GM-CSF) (Behnia et al., 2015). Therefore, senescence of amniotic membranes could make it difficult to maintain pregnancy, and delivery can be triggered by signals from aging foetal membranes. Apart from SASP-related molecules, DAMPs (HMGB1) (Menon et al., 2016; Plazyo et al., 2016) and heat shock protein (HSP) 70 (Chaiworapongsa et al., 2008; Chang et al., 2013; Dvorakova et al., 2017) are released by senescence-associated cellular injury from term membranes (Romero et al., 2011; Huang et al., 2015; Menon, 2016; Padron et al., 2020). The p38 MAPK signaling pathway associated with senescence and apoptosis have been shown to be activated by cellular injury (Bonney et al., 2016; Richardson et al., 2018; Menon et al., 2020), and the inflammation of foetal membranes can strengthen the senescent phenotype of membranes. All these changes can promote parturition.

Exosomes are lipid vesicles with double layers and a diameter of 30–150 nm that are secreted by cells, and their contents are mostly nucleic acids, lipids, proteins, microRNAs, etc., The contents carried by the secreted exosomes are different for different types and stages of cells (Abels and Breakefield, 2016). In recent years, studies on exosomes have shown that they are associated with inflammation, and inflammatory processes lead to many pathologic states, such as diabetes, arthritis, tumours, and neurodegenerative diseases, etc., Exosomes may be new biomarkers of inflammatory diseases according to the relationship between inflammation and exosomal cargo differences (Console et al., 2019). A study revealed that amniotic epithelial cell-derived exosomes can be taken up by myometrial, decidual and placental cells and can induce labour-associated inflammatory reactions in uterine cells. This result reveals that amniotic epithelial cell-derived exosomes may play a part in the delivery cascade by transmitting specific signals between the fetus and the mother (Hadley et al., 2018b). Exosomes, as vehicles that carry different cargos, can induce inflammation related to human spontaneous parturition (Menon et al., 2017; Mosaad et al., 2020). Other studies have shown that exosomes in amniotic fluid can pass through the placenta to reach the uterus, which provides strong evidence for exosomes as a medium for signal transduction (Sarker et al., 2014; Dixon et al., 2018).

Lungs produce pulmonary surfactant, a mixture of surfactant proteins (SP-A, SP-B, SP-C, and SP-D) and lipids that are the basis of respiration, and pulmonary surfactant is at the alveolar liquid gas interface and involved in normal physiological respiration (Guagliardo et al., 2018; Wang et al., 2020). During pregnancy, the alveoli gradually mature and surfactant protein levels increase, and some studies have revealed that pulmonary surfactant synthesis may be involved in the onset of labor (Montalbano et al., 2013; Mendelson et al., 2017). Animal experiments have verified that the increased production of pulmonary surfactant SP-A by the term fetal lung has an significant influence on the inflammatory cascade, which cause enhanced uterine contraction resulting in delivery (Condon et al., 2004a). Therefore, we proposed the following hypothesis: the occurrence of delivery is associated with amniotic membrane senescence, while the fetal lung maturation time is the closest to the time of term labor; thus, exosomes derived from the fetal lungs are released into the amniotic fluid through the swallowing behavior of the fetus in the third trimester of pregnancy that act on the amniotic membrane, which causes amniotic membrane aging, initiates apoptotic signaling pathway, induces the uterus to enter an inflammatory state and induces the occurrence of labor.

## Materials and Methods

### Amniotic Membrane Collection and hAEC Isolation

The ethics committee of Shandong Provincial Hospital has approved this experiment. Fresh placentas from full-term women were collected in the Obstetrics and Gynecology Department of Shandong Provincial Hospital. Hepatitis B and C, syphilis, HIV and fetal malformations were ruled out in the prenatal examination, and the pregnant women signed an informed consent form.

To explore whether the amniotic membrane ages at term labor, we obtained multiple samples from the rupture-zone, peri-placental zone not including the placenta and mid-zone areas from women who underwent spontaneous vaginal delivery at term (TL-AM) or elective cesarean delivery with no contraction (TNIL-AM) (McLaren et al., 1999; Nhan-Chang et al., 2010). And the senescence-associated-β-galactosidase (SA-β-gal) was performed to demonstrate cellular senescence of amniotic membrane bothTNIL-AM and TL-AM. For hAEC isolation, the amniotic membrane tissue was aseptically peeled off from the placenta, placed in sterile saline, and transported to the laboratory within 3 h. The isolation process was performed as previously described (Murphy et al., 2010). The membrane was fully rinsed with sterile saline until there was no blood attached to it. The amniotic membrane was cut into small 1 cm × 1 cm pieces, and all pieces of amniotic membrane were transferred into a sterile specimen container containing 0.05% trypsin in Hank’s solution (D-Hanks; Boster). After incubation for 60 min at 37 °C with 200 rpm shaking to digest the tissues, the digested cells were filtered with a 300-mesh cell strainer, and a same volume of DMEM (Gibco) complete medium containing 10% FBS (Gibco) was used to stop digestion. The cell suspension was centrifuged for 10 min at 1500 rpm, the supernatant was removed, and the sediment was resuspended in 1 ml of DMEM, including 10% exosome-free FBS, 1% penicillin and 100 μg/ml streptomycin (Solarbio). FBS without exosomes was obtained at 120,000×g for 18 h by ultracentrifugation (Shelke et al., 2014), and then, the supernatant was filtered through a 0.22-μm membrane (Millipore). Next, the cells were inoculated at a concentration of 1 × 10^5 into a T25 flask (Corning) and incubated at 37°C in 5% carbon dioxide (CO2) until the cell confluence reached 80–90%. The cell state was observed under a microscope (Olympus) and photographed and recorded every day.

### Immunofluorescence of hAEC

hAECs (1 × 10^4) were seeded into 96-well plates (Corning) and incubated for 4–5 h to allow the cells to adhere to the plate walls. Then, 4% paraformaldehyde was used to fix the cells for 15 min, and cells were permeabilized with 0.5% Triton X-100 for 20 min. Lastly, goat serum (Boster) was used to block the samples for 30 min. All procedures were performed at room temperature. hAECs were identified with a rabbit monoclonal primary cytokeratin 19 antibody (Abcam) diluted 1:200 in primary antibody dilution overnight at 4°C. Next, the plates were incubated with 1:500 Alexa Fluor 488-labeled goat anti-rabbit IgG (H + L) (Beyotime) for 1 h at room temperature, and nuclei were stained with DAPI (Solarbio). Confocal microscopy was applied for imaging.

### Human Lung Carcinoma Cell Line (A549) Culture and Supernatant Collection

The human lung carcinoma cell lines (A549) was purchased from Bluef (Shanghai) Biotechnology Development Co., Ltd. Cells were cultured in RPMI-1640 medium (Gibco) supplemented with 10% exosome-free FBS (Gibco), 1% penicillin and streptomycin (Gibco). Cell culture was maintained at 37°C in a humidified atmosphere with 5% CO2. After 48 h of culture, the medium in each well was collected and centrifuged instantly under the condition of 800×g for 10 min to isolate cell precipitations, and then, the supernatant was stored at −80°C until analysis.

### Amniotic Fluid Collection

Collection of amniotic fluid (AF) samples was carried out with the support of the ethics committee of Shandong Provincial Hospital, and informed consents were obtained from patients. AF samples were collected from women who underwent 1) spontaneous term labour without complications and vaginal parturition (TL; *n* = 10); spontaneous term labour without complications was defined as regular, progressively increasing uterine contractions lasting 30 s or more with 5–6 min intervals, accompanied by disappearance of the cervical canal, dilation of the cervix, and descent of the fetal presentation followed by labour at ≥ 37 gestational weeks and ≤42 gestational weeks with no pregnant complications during gestation, and 2) normal term not in labour (TNIL; *n* = 10), defined as opting for elective cesarean operation to delivery at 37–42 weeks of normal gestation. Exclusion criteria included preterm birth, post-term pregnancy, multiple gestations, gestational hypertension/preeclampsia, placental abruption/previa, infant deformities, gestational diabetes, and intrauterine growth restriction.

For vaginal labour, AF samples were suctioned by using a 10-ml syringe through the dilated cervix before artificial rupture of membrane. For elective cesarean delivery, after exposure of the integral amniotic sac, a 10-ml syringe was used to collect AF samples to avoid contamination. Sediments in all AF samples were immediately removed by centrifuging under the condition of 2000×g for 10 min. After that, the AF supernatant was stored at −80°C in the dark until analysis.

### Exosome Isolation From AF and A549 Cell Supernatant

Exosomes were isolated from AF and A549 cell supernatants as previously described (Dixon et al., 2018; Sheller-Miller and Menon, 2020). In brief, an equal volume of 1× phosphate-buffered saline (PBS) (Solarbio) was used to dilute the AF sample, which was centrifuged at 300×g for 10 min at 4°C. Then, the supernatant was centrifuged at 2,000×g for 30 min at 4°C. The resultant supernatant fluid was then centrifuged at 12,000×g for 45 min at 4°C. The sediment was discarded, and the supernatant was filtered through a 0.22-µm filter (Millipore). Next, the filtered fluid was transferred into an ultracentrifuge tube, and ultracentrifugation (Hitachi) was performed at 120,000×g for 70 min twice. The pellet containing the small vesicles was resuspended in 200 µl of 1× PBS and stored at −80°C. The above steps for AF exosome isolation are also suitable for exosomes derived from A549 cell supernatant.

### Animals

C57BL/6 (B6) mice were purchased from Jinan Pengyue Laboratory Animal Breeding Co., Ltd., and were bred in the animal care facility at the animal laboratory of Shandong Provincial Hospital. All mice were raised under a circadian rhythm condition (dark time: light time = 12:12). Eight-week-old females, after adaptive feeding for 1 week, were mated with males of the same phenotype. From 8:00 a.m. to 9:00 a.m. on the second day, we checked daily for the appearance of a vaginal plug indicating 0.5 days post-coitum (dpc). Females were then separated from the males, and their body weight was monitored every day. Until 12.5 dpc, if a mouse gained two or more grams until 12.5 dpc, it was considered pregnant. All animal experiments has approved by the Ethics Committee of Shandong Provincial Hospital.

### Transmission Electron Microscopy

For assessment of exosome morphology, exosomes were resuspended in 50 µl of 1× PBS solution, and a drop of the solution was loaded onto a copper grid at room temperature for 10 min. Then, 3% glutaraldehyde was used for fixation for 5 min, and excess glutaraldehyde was blotted off with filter paper. The exosomes were then transferred to diluted water solution for 2 min, and this step was repeated 10 times. Finally, 4% uranyl acetate was used for washing. Grids were allowed to dry for approximately 30 min and subsequently imaged in a transmission electron microscope in the laboratory of Shandong Second Provincial General Hospital.

### Nanoparticle Tracking Analysis

Exosomes were resuspended in 50 µl of 1× PBS, and 5 µl of resuspended solution was used for NTA. NTA measurements were performed using a ZetaView® Nanoparticle Tracking Analyzer PMX-120 following the manufacturer’s instructions. First, the instrument performance was tested, and then the samples were examined. From each sample, the particle size and concentration information can be obtained with the outer probe of the instrument.

### Western Blot (CD63, TSG101, EpCAM, and SP-C)

Study has verified that extracellular vesicle (exosme) produced upon fusion of endosomal multivesicular bodies (MVBs) with the cell membrane, and CD63 is a tetraspanin which is necessary for this process. So exosome contains a higher concentration of CD63 (Mathieu et al., 2021). TSG101 is one of the main component of the endosomal sorting complex required for transport (ESCRT) being involved in MVB and exosome biogenesis, and TSG101 is detected in purified exosomes from heterogeneous cell types by proteomic analysis (Colombo et al., 2013). Alveolar epithelial type II cells (AT2) can synthetize pulmonary surfactant, and a common way to identify AT2 cells is to use SP-C which is the only AT2-specific protein (Beers and Moodley, 2017). EpCAM is a pan-epithelial cell marker, and it can be detected on the surface of AT2 (Hasegawa et al., 2017). The expression of exosomal CD63 and TSG101 and the alveolar epithelial type II cell markers EpCAM and SP-C were evaluated by western blot analysis. Exosomes isolated from amniotic fluid (AF-exos) and A549-exos were lysed using a precooled RIPA and PMSF mixed buffer (100:1) and centrifuged for 30 min at 4 °C. The exosomal protein concentration was quantified by a BCA Protein Assay Kit.

Samples were loaded on a 10% SDS–PAGE gel and then transferred onto PVDF membranes. Then, 5% skim milk dissolved in 1× TBST was used to block the membranes for 1 h at room temperature, and the membranes were probed with anti-CD63 (1:1000; Abcam), anti-TSG101 (1:1000; Abcam), anti-SP-C (1:1000; Sigma) and anti-EpCAM (1:1000; Proteintech) primary antibodies at 4°C overnight. After the membranes were washed three times with Tris-buffered saline with Tween (TBST) buffer, they were incubated with secondary horseradish peroxidase (HRP)-conjugated anti-rabbit antibody (Solarbio) at room temperature for 1 h. The blots were washed with TBST for three times, and the Amersham Imager 600 Imaging System (GE Healthcare, Chicago, IL) was used for imaging.

### Exosome Labeling

Fluorescently detectable AF-exos and A549-exos were generated by using PKH67 green membrane dye (Sigma). Hoechst and phalloidin were used to stain the cell nucleus and cytoskeleton, respectively. hAECs grown to approximately 80–90% confluency were washed twice with 1× PBS and cultured in a 96-well plate with medium containing 10% exosome-free FBS for 24 h. Briefly, the staining steps were as follows: 20 µl of exosomes was added to 500 µl of Diluent C (solution A) and 2 µl of PKH67 dye mixed with 500 µl of Diluent C (solution B) in the dark. Then, solutions A and B were mixed quickly and incubated for 5 min at room temperature in the dark. Next, 1 ml of 1% bovine serum albumin (BSA; Solarbio) was added to stop the labeling. The stained exosomes were resuspended in 1× PBS buffer and then ultracentrifuged at 110,000×g for 70 min at 4°C. The supernatant was eliminated, and the PKH67-labeled exosomes were resuspended in 130 μl of 1× PBS. Ten microliters of labeled exosomes was added to each well and incubated overnight at 37°C and 5% CO2. The next day, 100 µl of 4% paraformaldehyde (Solarbio) was added to each well to fix at room temperature for 15 min, followed by washing three times with 1× PBS. Then, each well received 100 µl of cell membrane permeabilization solution (Solarbio) at room temperature for 5 min. Every well was washed three times with 1× PBS, followed by the addition of 70 µl of phalloidin to each well and incubation of the plate in the dark and at room temperature for 30 min. After that, 100 µl of Hoechst was added to each well and, the samples were incubated for 5 min in a dark place. Finally, the samples were imaged using ImageXpress Micro Confocal with MetaXpress software.

### HAEC Treatment With Exosomes

For the AF-exos groups, 100 µg of TNIL-exos and 100 µg of TL-exos were dissolved in 1 ml of complete DMEM containing 5% exosome-free FBS and filtered through a 0.22-µm membrane. HAECs were cultivated in 6-well plates, and the above medium was used to culture the hAECs for 24 h.

For the A549-exos groups, 100 µg of A549-exos was added to 1 ml of DMEM complete medium including 5% exosome-free FBS as the experimental group and filtered. Complete DMEM (1 ml) containing 5% exosome-free FBS was used as the control group. The two kinds of medium were used to culture hAECs seeded in 6-well plates for 24 h.

### Western Blot

After coincubation with TNIL-exos, TL-exos and A549-exos for 24 h, the total protein was extracted from cells, and the same amount of protein was electrophoresed on SDS-PAGE. A PVDF membrane was used to transfer the protein, followed by incubation in 5% skim milk to block for 1 h. Then, the primary antibodies anti-p38 MAPK (1:1000, Abcam), anti-pp38 MAPK (1:1000, Abcam), anti-HSP70 (1:1000, Abcam) and anti-HMGB1 (1:1000, Abcam) were added and incubated at 4°C overnight. Tubulin (1:1000, Abcam) served as the internal controls. The goat anti-rabbit and goat anti-mouse antibodies (1:1000, Solarbio) conjugated with HRP were used as secondary antibodies. Chemiluminescence detection was performed using the Amersham Imager 600 Imaging System.

### SA‐β‐gal Assay

All experimental reagents used for amniotic membrane staining were purchased from Servicebio company. First, the amniotic membrane was cut into slices using a freezing microtome (Thermo). Then, the working solution was prepared, the fresh slices were fixed, β-galactosidase staining was performed, the slices were dehydrated and sealed, and the senescent blue cells were observed under a microscope.

### Enzyme-Linked Immunosorbent Assay for SASP Markers

The supernatant of hAECs cocultivated with TNIL-exos, TL-exos and A549-exos for 24 h was centrifuged at 1000×g for 15 min before use and stored at −80°C. Concentrations of the SASP markers MMP9, GM-CSF, TNF-a, IL-6 and IL-8 were measured by ELISAs (Cusabio Biotech). The experimental process was performed following the manufacturer’s protocol. Briefly, all reagents, working solution, standard solution and samples were prepared as instructed, 100 μl of standard or sample was added to each well, and the plates were incubated for 2 h at 37°C. Then, 100 μl of biotin-antibody (×1) was added to each well, incubated for 1 h at 37°C, aspirated from each well and washed, and 100 μl of HRP-avidin (×1) was added to each well. The plates were incubated for 1 h at 37°C, and 90 μl of TMB substrate was added to each well and kept for 15 min at 37°C protected from light. Finally, each well was filled with 50 μl of stop solution, the plate was gently shaken to ensure thorough mixing, and a microplate reader (BioTek; United States; IL) was applied to measure the absorbance at 450 nm.

### Apoptosis Assay

HAECs were cocultured with TNIL-exos, TL-exos and A549-exos for 24 h. According to the instruction of the apoptosis kit (BD Biosciences), an apoptosis assay was performed. Briefly, the medium was removed, and the resuspended cell solution had a concentration of 1 × 10^6 cells/ml. Then, the staining solution was mixed with 100 µl of cell suspension in 1.5-ml EP tubes for 15 min in the dark. Next, 500 µl of binding buffer was added and mixed on ice. Flow cytometry (BD Accuri™ C6 Plus) was used to detect apoptotic cells within 1 h.

### Intraamniotic Administration of A549-Derived Exosomes

Pregnant B6 mice were anesthetized at 14.5 dpc by intraperitoneal administration of 50 µl of propofol. Mice were placed on an operation table and stabilized with tape. After disinfection with iodophor, we used sterile scissors to open the abdomen of the mice and recorded the number of fetuses. A concentration of 500 µg of A549-exos dissolved in 50 µl of sterile PBS was injected into each amniotic cavity using a single-use insulin syringe (*n* = 12). Controls (*n* = 12) were treated with an equal volume of sterile 1× PBS alone (Radnaa et al., 2021). After injection with exosomes, the mice were monitored every morning (8:00 a.m.) before 19.5 dpc to determine vaginal bleeding before preterm birth (≤18.5dpc) and the rate of preterm birth. Another pregnant mouse was injected with PKH 67-labeled A549-exo at 14.5 dpc through the same way, and sacrificed by cervical dislocation after 24 h to obtain the amniotic membranes and placentas.

### Isloation of Amnion and Placenta-Derived Cells

The intact amniotic sacs were collected by removing uterine tissue, the amniotic membranes and placentas were collected respectively. The membranes were cut into small pieces and all pieces of membranes were digested with 0.25% trypsin. After incubation for 45 min at 37°C, the digested cells were filtered with a 300-mesh cell strainer, and a same volume of DMEM/F12 complete medium containing 10% FBS was used to stop digestion. The cell suspension was centrifuged for 5 min at 1500 rpm, the supernatant was removed, and the sediment was incubated in red blood cell (RBC) lysis buffer. Then the cell suspension was centrifuged for 5 min at 1500 rpm, and the cell pellets were resuspended in 1 ml 1× PBS to remove the excess RBC lysis buffer. Lastly, the 1 ml completed medium of DMEM/F12 was used to resuspend the obtained cells followed by seeding into 96-well plates and incubating until the cells adhered to the plate walls. The placenta tissue was cut into 1 mm^3^ pieces and digested with collagenase Type IV at 37°C for 30 min. The following steps were the same with the process of amniotic cells isolation.

### Immunofluorescence of Amnion and Placenta-Derived Cells

This procedure was performed to demonstrate that whether the PKH67-labeled exosomes can be taken up by amniotic cells as well as placental derived cells or not. After the isolated cells adhered to the wall, 4% paraformaldehyde was used to fix the cells for 15 min, and followed by cell nuclei staining with DAPI for 8 min. All procedures were performed at room temperature in the dark. Fluorescence microscope was applied for imaging.

### Statistical Analysis

Data are showed as the mean ± SD. The ImageJ software was used to analyse the western blot and immunofluorescence results, the GraphPad Prism version 7 was applied to perform the statistical analyses. The analyses of vaginal bleeding and preterm birth relied on Fisher’s exact test, and other statistical analyses were performed with Student’s t test. A *p* value <0.05 was defined as significant.

## Results

### SA-β-gal of the Amniotic Membrane and Characterization of hAECs

**FIGURE 1:**
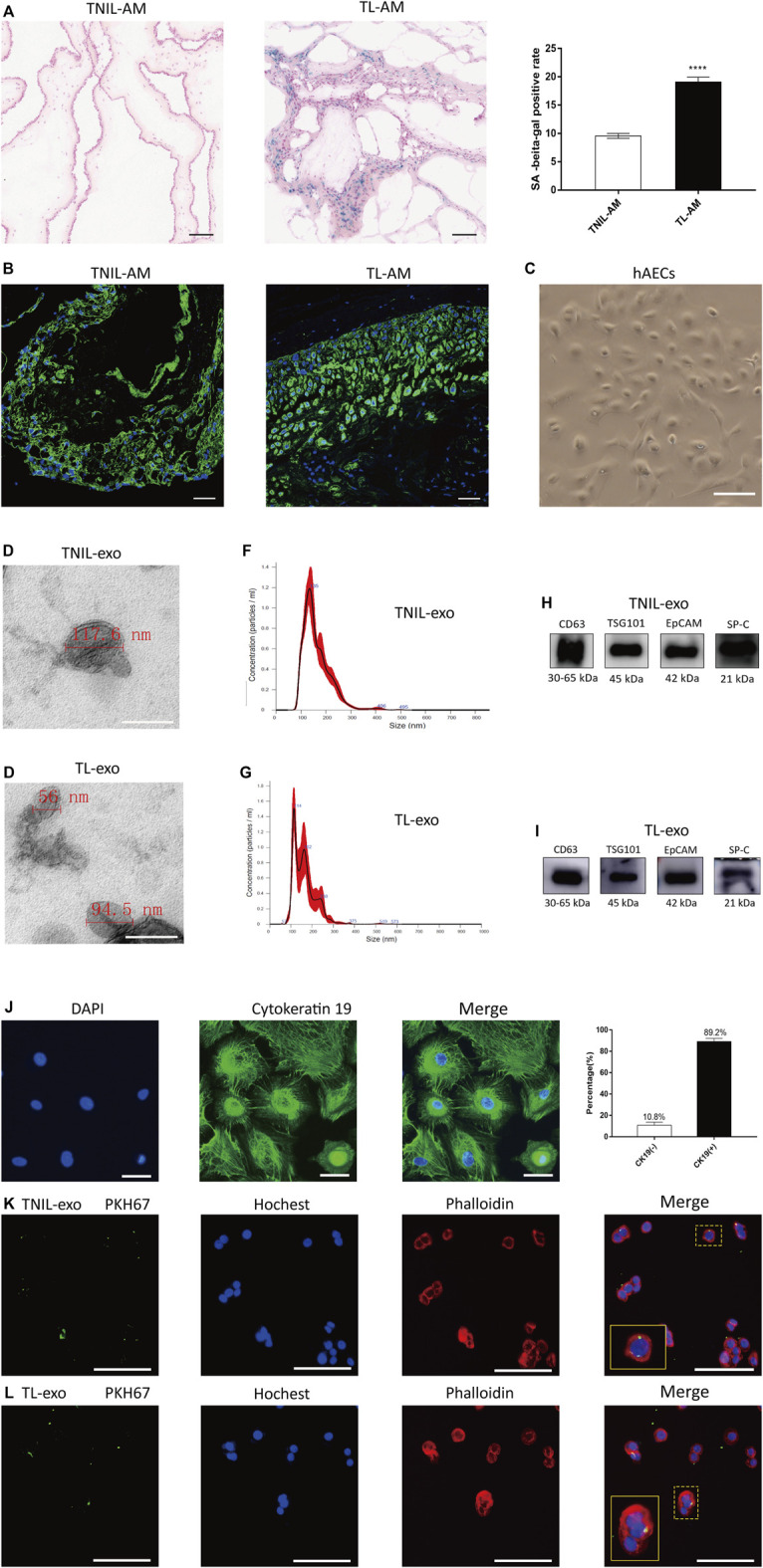
Amniotic membrane staining and characteristics of AF-exos. **(A)**: The SA-β-gal staining of amniotic membrane both from TNIL-AM and term labor TL-AM. The latter showed a higher positive staining rate and revealed that TL-AM is associated with cellular senescence. Scale bar, 50 µm. **(B)**: The CK 19, marker of epithelial cells, was positive in all membrane tissue. Blue: nucleus; green: cytokeratin 19; scale bar: 50 µm. **(C)**: The morphology of primary hAECs under an inverted microscope after 7 days of growth; scale bar, 200 µm. **(D,E)**: The electron microscopy showed the cup‐shaped morphology of TNIL-exo and TL-exo; scale bar = 200 nm. **(F,G)**: Nanosight analysis of TNIL-exo and TL-exo all showed a single peak at 50∼150 nm. **(H,I)**: Western blot revealed TSG101 and CD63 are representative markers of exosomes, and EpCAM and SP-C are markers of alveolar type II epithelial cells. The four markers were positively expressed by TNIL-exo and TL-exo. **(J)**: The marker of epithelial cells (cytokeratin 19) positive staining in isolated cells and statistics analysis, blue: nucleus; green: cytokeratin 19; scale bar: 50 µm. **(K,L)**: Uptake of TNIL-exo and TL-exo in hAECs was observed by confocal fluorescence microscopy; green: exosomes; blue: nucleus; red: cytoskeleton; scale bar = 100 µm.

Under a light microscope, the TL-AM showed a higher positive staining rate than that of TNIL-AM (Figure 1A). The staining of cytokeratin 19 both TNIL-AM and TL-AM were all positive (Figure 1B). The 7-day hAECs appeared as typical pebble-shaped cells (Figure 1C). The cytokeratin 19 antibody, a marker of epithelial cells, was used to determine the purity of hAEC culture. Green fluorescence intensity could be observed, and the statistics analysis revealed a pretty high percentage of hAECs, which suggested that the about 90% isolated cell were hAECs (Figure 1J).

### Characterization and Internalization of Exosomes Derived From AF

Exosomes were isolated as previously described, and PKH67-labeled TNIL-exos and TL-exos were taken up by hAECs (Figure 1K,L). Both TNIL-exo and TL-exo groups showed the presence of typical cup-shaped vesicles by electron microscopy (Figures 1D,E). NTA indicated that the diameter of the two kinds of exosomes were between 50 and 150 nm (Figures 1F,G). Western blotting identified exosome surface protein markers (CD63 and TSG101) and alveolar epithelial type II cell surface markers (EpCAM and SP-C, Figures 1H,I).

### TL-Exos Induced p38 MAPK Activation and DAMP Release

**FIGURE 2:**
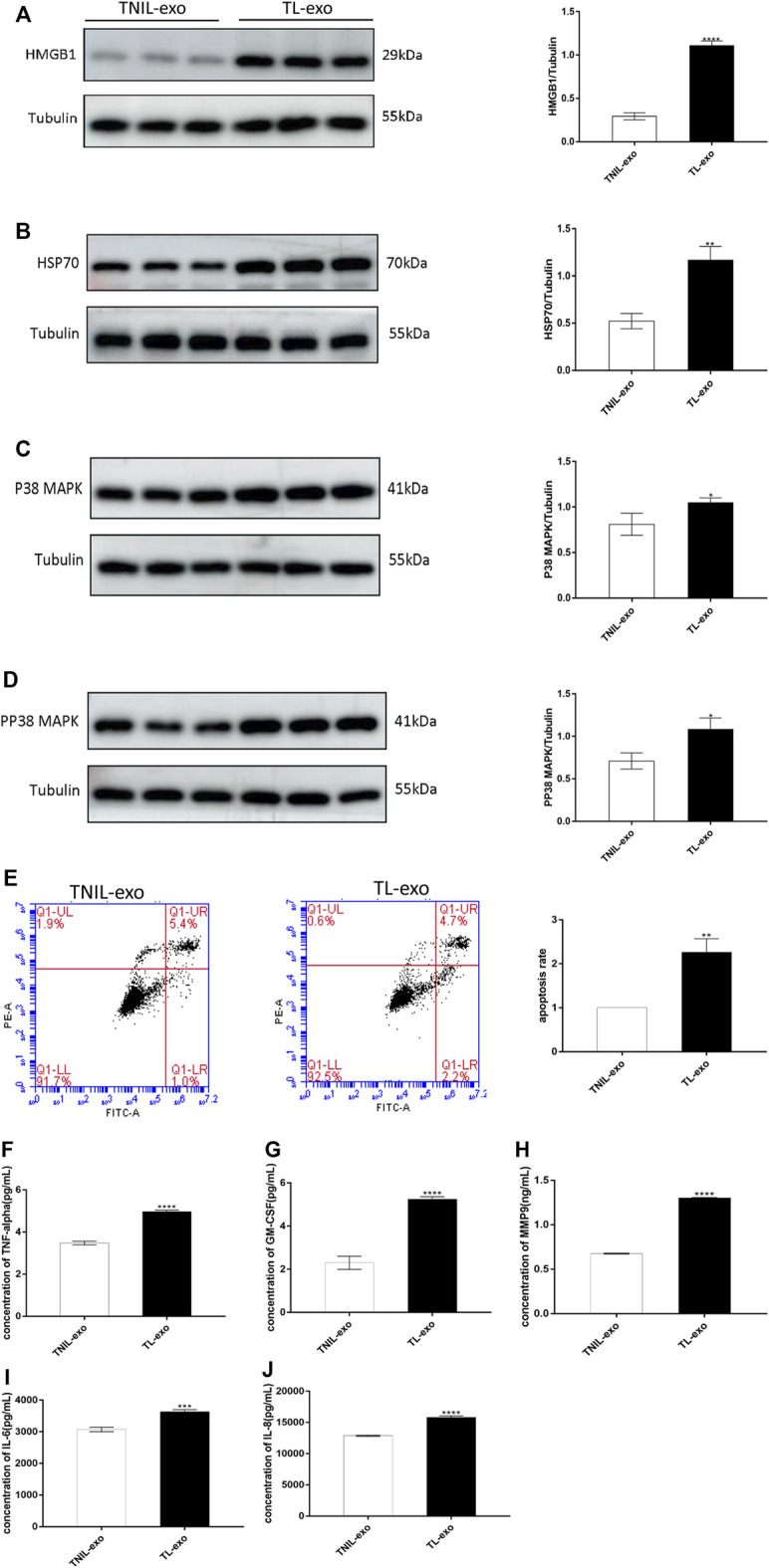
TL-exos induced hAEC aging and apoptosis, activated senescence-associated signaling pathway and released inflammatory cytokines. **(A)**: Western blot of HMGB1 protein expression in hAECs of TNIL-exos and TL-exos groups; **(B)**: Western blot of HSP70 protein expression in hAECs of two groups; **(C)**: Western blot of p38 MAPK protein expression in hAECs of two groups. **(D)**:Western blot of pp38 MAPK protein expression in hAECs of two groups. The levels of four proteins were higher in the TL-exos group than that in the TNIL-exos group. **(E)**: Cellular apoptosis was assessed using flow cytometry: TL-exos induced more hAECs apoptosis and senescence. The concentration of SASP molecules (MMP9, TNF-α, IL-6, GM-CSF and IL-8) released by hAECs in the TNIL-exos and TL-exos groups. **(F)**: Concentration of TNF-α; **(G)**: Concentration of GM-CSF; **(H)**: Concentration of MMP9; **(I)**: Concentration of IL-6; **(J)**: Concentration of IL-8. The ELISA results showed that TL-exos induced inflammation in hAECs and the release of more SASPs than TNIL-exos. All data are presented as the mean ± SD. ∗*p* < 0.05, ∗∗*p* < 0.01, ∗∗∗*p* < 0.001, Student’s t test. All experiments were performed in triplicate.

P38 MAPK is a member of the MAPK family and is known to play a significant role in cellular inflammation, aging and apoptosis. Therefore, activation of p38 MAPK can induce cell senescence and apoptosis, and the initiation of cell injury results in the release of DAMPs, which cause innate immune and inflammatory cascades reactions by pattern recognition receptors. In this experiment, western blot analysis revealed that the protein levels of DAMPs (HSP70 and HMGB1) were higher in the TL-exos group (Figures 2A,B). The levels of p38 MAPK and pp38 MAPK were higher than those in the TNIL-exos group (Figures 2C,D), which showed that the senescence-associated signaling pathway of hAECs were activated after treatment with TL-exos.

### TL-Exos Caused hAEC Apoptosis

Flow cytometry suggested that after TL-exos intervention, hAECs had a more obvious apoptosis rate than TNIL-exos-treated hAECs, showing a pro-apoptotic effect of TL-exo (Figure 2E).

### TL-Exos Induced hAEC Inflammation

SASP markers include matrix metalloproteinases (MMPs), cytokines, growth factors, chemokines, and enzymes generating prostanoids, and they are released by senescent foetal amniotic membrane cells. In this experiment, hAECs treated with TL-exos displayed higher levels of SASPs including MMP9, TNF-α, GM-CSF, IL-6, and IL-8 than those of the control group (Figures 2F–J).

### Characterization and Internalization of A549-Exos

**FIGURE 3:**
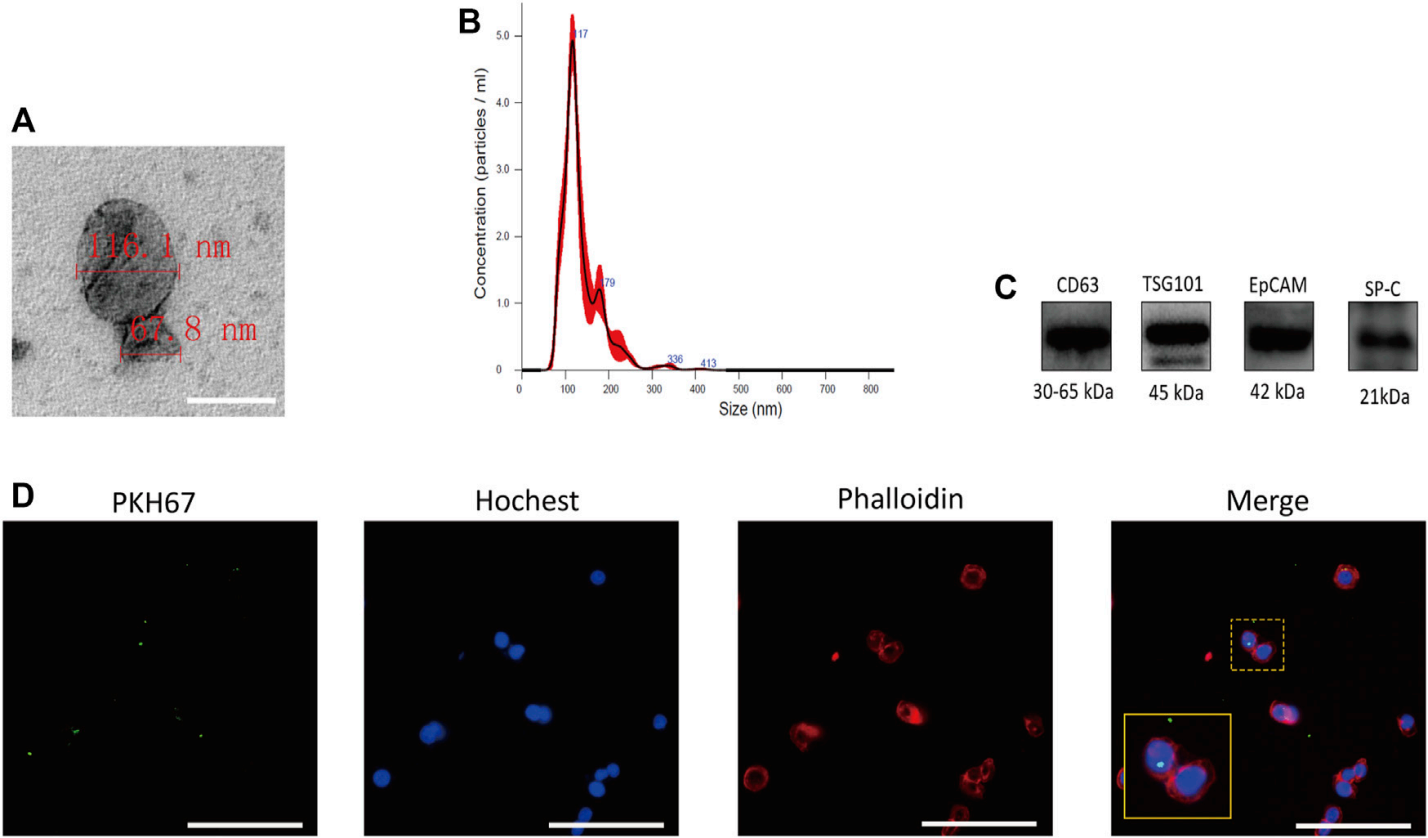
Characteristics of A549-exos. **(A)**: Electron microscopy suggesting the cup‐shaped morphology of exosomes; scale bar = 200 nm. **(B)**: Nanosight analysis of A549-exos showed a single peak at 50∼150 nm. **(C)**: TSG101 and CD63 are representative markers of exosomes, and EpCAM and SP-C are markers of alveolar type II epithelial cells. The four markers were positively expressed by A549-exos. **(D)**: Uptake of A549-exos in hAECs was observed by confocal fluorescence microscopy; green: A549-exos; red: cytoskeleton; blue: nucleus; scale bar = 100 µm.

A549-exos were isolated as previously described, and electron microscopy showed the presence of representative cup-shaped vesicles with diameters ranging from 50 to 150 nm (Figure 3A). NTA showed that particles between 50 and 150 nm were obtained (Figure 3B) and positively expressed CD63, TSG101, EpCAM, and SP-C (Figure 3C). After incubation with hAECs overnight, A549-exos were taken up by hAECs (Figure 3D). Neither AF-exos nor A549-exos showed obvious differences in morphology, diameter, surface markers or the process of internalization.

### A549-Exos Cause Proapoptotic p38 MAPK Signaling Pathway Activation and DAMP Release

**FIGURE 4:**
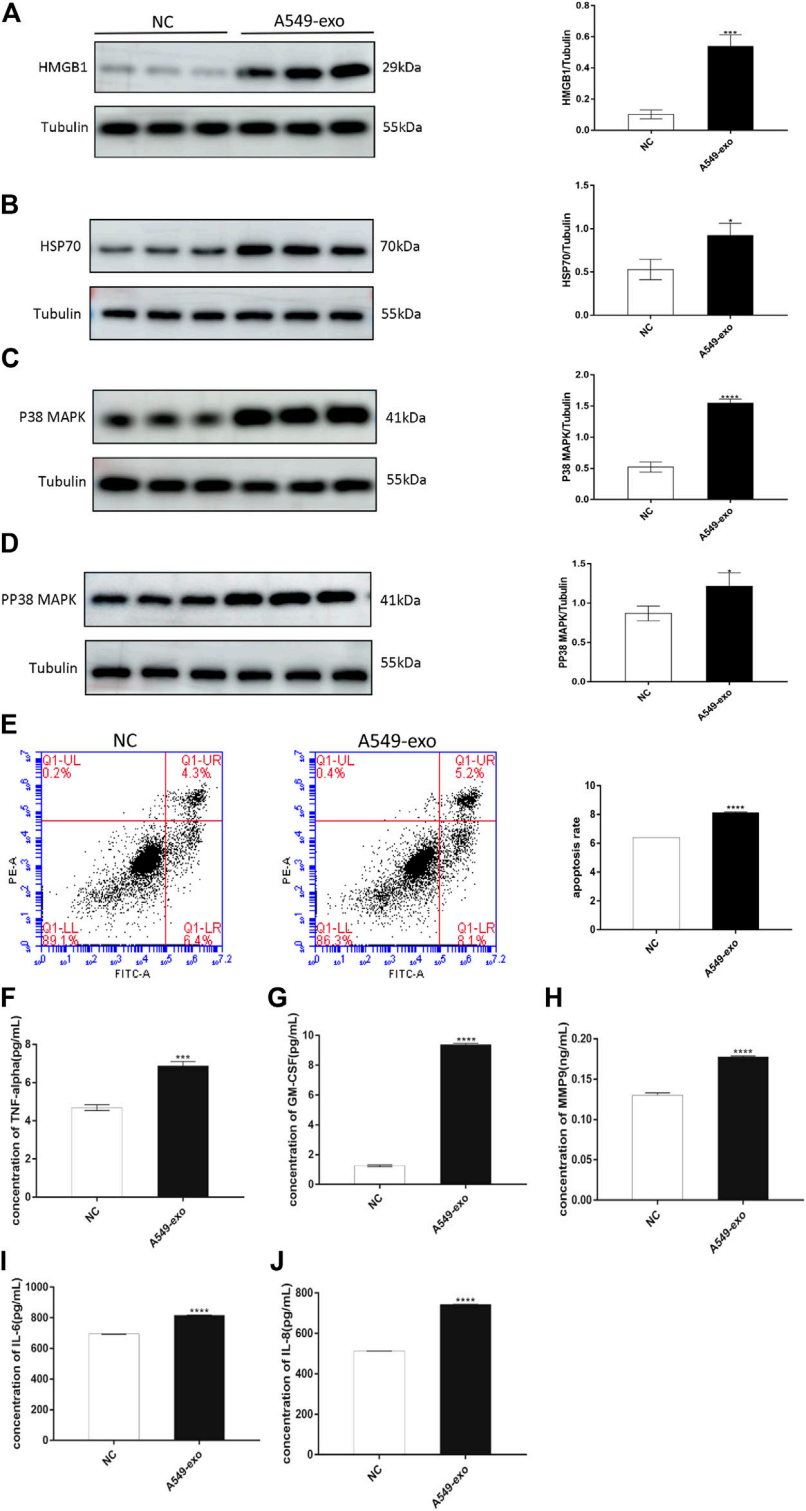
A549-exos cause hAECs to undergo aging and apoptosis, to activate senescence-associated signaling pathway and to release inflammatory cytokines. **(A)**: Western blot of HMGB1 protein expression in hAECs; **(B)**: Western blot of HSP70 protein expression in hAECs; **(C)**: Western blot of p38 MAPK protein expression in hAECs; **(D)**: Western blot of pp38 MAPK protein expression in hAECs. All target protein levels were higher in the A549-exos group than in the control group. **(E)**: Apoptosis was assessed using flow cytometry: A549-exos induced more hAEC apoptosis and senescence. **(F)**: Concentration of TNF-α; **(G)**: Concentration of GM-CSF; **(H)**: Concentration of MMP9; **(I)**: Concentration of IL-6; **(J)**: Concentration of IL-8. The ELISA results showed that the A549-exos induced inflammation in hAECs and the release of more SASPs than the control. All data are presented as the mean ± SD. ∗*p* < 0.05, ∗∗*p* < 0.01, ∗∗∗*p* < 0.001, Student’s t test. All experiments were performed in triplicate.

The roles of the p38 MAPK pathway in senescence and apoptosis have been described above, and we also already know that DAMPs are released by injured and senescent cells. The subsequent western blot analysis showed that the protein levels of pp38 MAPK, p38 MAPK and DAMPs were higher in the A549-exos group than in the control group (Figures 4A–D). Both the AF-exos and A549-exos groups’ results revealed that exosomes from term labor amniotic fluid may induce amniotic membrane senescence.

### A549-Exos Cause Cell Apoptosis and the Release of SASPs

The definition of SASPs has already been introduced, and the representative markers include MMP9, TNF-α, GM-CSF, IL-6, and IL-8. After cocultured with A549-exos, the hAEC supernatant had a higher concentration of SASPs than that of the NC group (Figures 4F–J).

### A549-Exos can Be Taken up by the Amniotic Membrane and Placenta and Induce Preterm Birth

**FIGURE 5:**
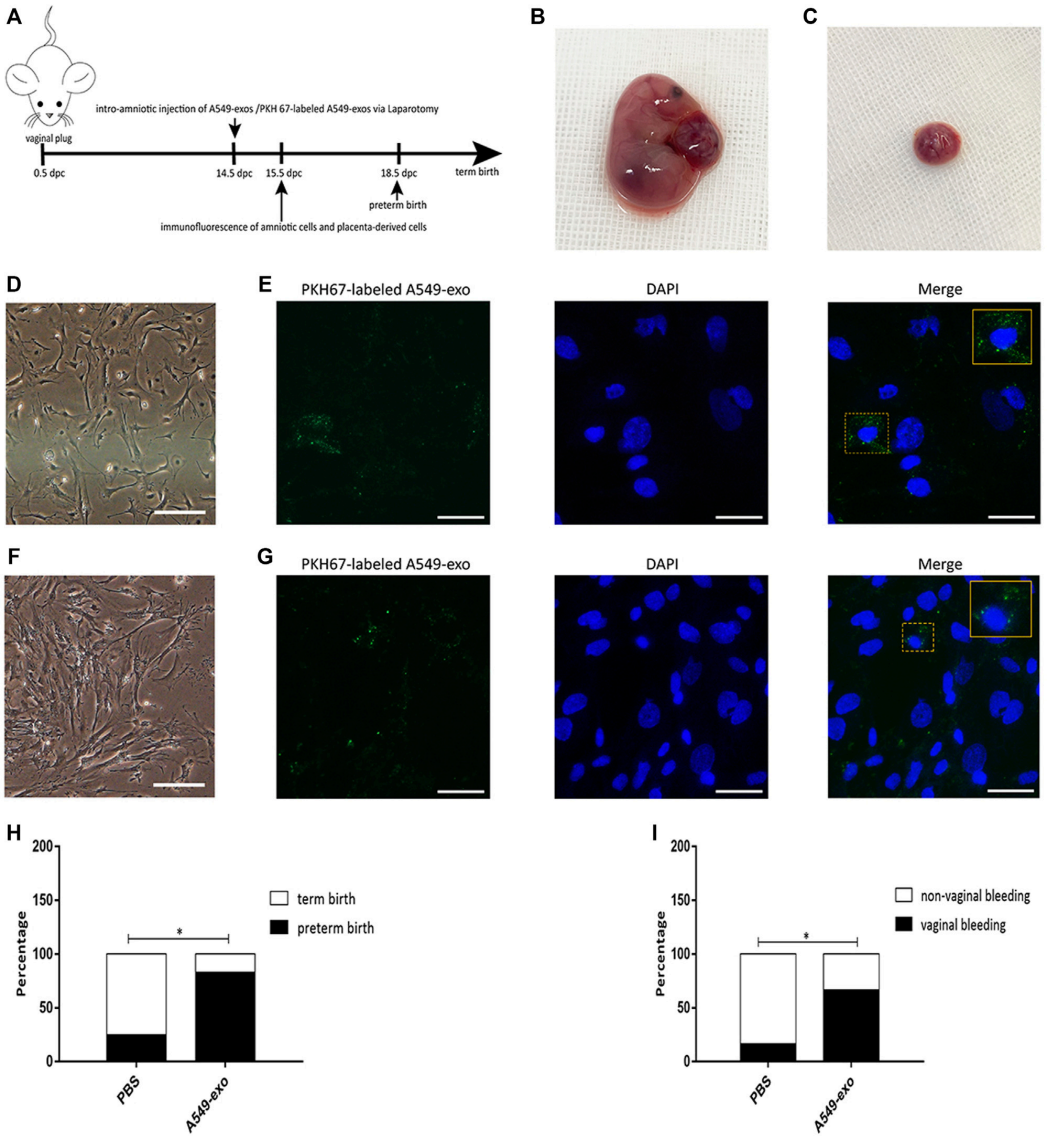
Animal experiment. **(A)**: the timeline of experiment. At 14.5 dpc, pregnant mice were injected with A549-exos or PKH67-labeled A549-exos via the intraamniotic route (*n* = 12) or 1× PBS (*n* = 12). **(B)**: Intact amniotic sac. **(C)**: The placenta. **(D)**: The morphology of amniotic cells under an inverted microscope; scale bar, 200 µm. **(E)**: The PKH67-labeled A549-exo was detected around the nucleus of amniotic cells; green: exosomes; blue: nucleus; scale bar, 50 µm. **(F)**:The morphology of placenta-derived cells under an inverted microscope; scale bar, 200 µm. **(G)**:The PKH67-labeled A549-exo was detected around the nucleus of placenta-derived cells; green: exosomes; blue: nucleus; scale bar, 50 µm. **(H)**: Rate of vaginal bleeding before preterm birth between the two groups (≤18.5 dpc). **(I)**: Rate of preterm birth between the two groups.

After 24 h, the intact amniotic sacs and placentas of PKH67-labeled A549-exo-injected mouse were all collected (Figures 5B,C). The cell morphology of amniotic cells and placenta-derived cells was different (Figures 5D,F) and the labeled A549-exos were observed around the nucleus, which demonstrated that exosome can be taken up by amniotic membrane and placenta (Figures 5E,G). The frequency of vaginal bleeding was higher after intraamniotic injection of A549-exos than PBS [A549-exos 66.7% (8/12) versus PBS 16.7% (2/12); *p* = 0.0361; Figure 5H]. Apart from that, intraamniotic injection of A549-exos also showed an increased rate of preterm birth compared with that of PBS [A549-exos 83.3% (10/12) versus PBS 25% (3/12); *p* = 0.0123; Figure 5I].

## Discussion

Studies have shown that the mechanisms of parturition vary, but the main mechanism of delivery is believed to involve the inflammation theory.

The concentrations of GM-CSF, IL-6, and IL-8 were higher in the term labour group, and the 3 kinds of proinflammatory cytokines were involved in the SASP (Behnia et al., 2015). At the end of pregnancy, oxidative stress reactions can lead the uterine cells to transit from the quiescent stage to the active phase. The change can stimulate uterine cells to produce labour-associated inflammatory cytokines which are prerequisites for parturition (Christiaens et al., 2008). The p38 MAPK signalling pathway is activated by cellular senescence, and DAMPs are also expressed at higher levels in aging tissue (Huang et al., 2015; Lavu et al., 2019). All of these results are consistent with our study. However, there are still some differences in previous studies compared with our research. Some researchers believe that the steady-state levels of progesterone receptor (PR)-A and trans inhibitory activity in the myometrium of the uterus was increased by proinflammatory stimuli, and functional progesterone withdrawal mediated by PR-A could induce parturition (Peters et al., 2017; Patel et al., 2018). There are still many theories about the rupture of foetal membranes. With regard to full-term or preterm pregnancies, there is a significant relationship between parturition and an increased level of proinflammatory cytokines of the amniotic membranes with or without infection, and activated MMPs are believed to be the reason behind the rupture of membranes. Another opinion is that IL-1β decreases the lysyloxidase (LOX) expression level, promoting phosphorylation of GATA3 and the NF-kB subunit p65, and the whole process depends on activating the p38 and Erk1/2 MAPK signal pathways, which cause the rupture of membranes (Zhang et al., 2017). An animal model also revealed some new insights: cell-free foetal DNA (cffDNA) can activate a nonspecific immune response, inducing the onset of delivery. The process is as follows: DNA depletion of telomeric sequences originating from mouse foetuses and placentas can stimulate Toll-like receptor 9 (TLR9), resulting in activation of macrophages, and the latter can secrete abundant proinflammatory cytokines involving the occurrence of labour (Goldfarb et al., 2018).

For our study, according to the positive SA-β-gal staining results of the amniotic membrane at term labor, we hypothesized that exosomes from term labor amniotic fluid may influence the induction of parturition. Therefore, we isolated TL-exos and TNIL-exos and separated hAECs from the amniotic membrane. After coculturing with exosomes from amniotic fluid, the hAECs of the TL-exos group showed a higher cell apoptosis rate than that of the TNIL-exos group. We also observed that TL-exos caused cell aging by activating senescence-associated p38 MAPK signaling pathway; DAMPs and SASPs displayed higher levels in the TL-exos group. The above results may serve as an explanation for term parturition. Because the literature has already verified that pulmonary surfactant may be associated with parturition (Condon et al., 2004b; Montalbano et al., 2013; Mendelson et al., 2017), we also speculated that since the foetal lung maturation time is the closest to the time of term labour, the exosomes derived from the foetal lungs are released into the amniotic fluid through the swallowing behaviour of the fetus in the third trimester of pregnancy and can act on the amniotic membrane. Exosomes from term fetal lungs resulted in amniotic membrane aging and induced labor. Therefore, we performed a series of experiments to confirm the second hypothesis. We purchased the human carcinoma A549 cell line as a model of alveolar epithelial type II cells. The former showed typical morphology and ultrastructural features, such as the lamellar body, which is responsible for pulmonary surfactant synthesis and release (Li et al., 2019; Nova et al., 2020). The A549 cell supernatant was collected and used to isolate A549-exos. After incubation with hAECs, we performed western blotting flow cytometry and ELISA, and all results of the A549-exos were consistent with previous TL-exos experimental results. Lastly, we performed animal experiments, and the results suggested that A549-exos can induce preterm birth, which provides evidence that pulmonary surfactant may play a significant role in laboring. In conclusion, our results suggested that exosomes derived from fetal lungs in term amniotic fluid may activate the senescence-associated p38 MAPK signaling pathway, cause hAEC aging and release DAMPs and SASPs, which serve as prerequisites for the start of term parturition. However, there are still some limitations of this study. First, we are unable to explain why TNIL-exos in the amniotic fluid cannot activate the signaling pathway, induce hAEC senescence and release more DAMPs and SASPs. Second, we cannot directly isolate exosomes in amniotic fluid from term fetal lungs and perform the following series of experiments, so the results may be affected. Therefore, future work should emphasize the direct isolation of fetal lung-derived exosomes to more definitively demonstrate that exosomes can induce fetal membrane senescence, which contributes to term labor.

## Conclusion

Fetal lung-derived exosomes of term labour in the amniotic fluid can induce amniotic membrane senescence and activate proapoptotic p38 MAPK signalling pathway, causing senescent hAECs to release senescence-associated secretory phenotype-related molecules and damage-associated molecular patterns, which may all contribute to term parturition.

## Data Availability

The original contributions presented in the study are included in the article/supplementary material, further inquiries can be directed to the corresponding authors.
